# Loss of STAT5A promotes glucose metabolism and tumor growth through miRNA‐23a‐AKT signaling in hepatocellular carcinoma

**DOI:** 10.1002/1878-0261.12846

**Published:** 2020-11-22

**Authors:** Yabo Jiang, Yongzhen Tao, Xiuping Zhang, Xubiao Wei, Min Li, Xuxiao He, Bin Zhou, Weixing Guo, Huiyong Yin, Shuqun Cheng

**Affiliations:** ^1^ The Six Department of Hepatic Surgery Eastern Hepatobiliary Surgery Hospital Second Military Medical University Shanghai China; ^2^ Key Laboratory of Nutrition, Metabolism and Food Safety Shanghai Institute of Nutrition and Health (SINH) Shanghai Institutes for Biological Sciences (SIBS) Chinese Academy of Sciences (CAS) Shanghai China; ^3^ Department of Hepatobiliary and Pancreatic Surgical Oncology The First Medical Center of Chinese People’s Liberation Army (PLA) General Hospital Beijing China

**Keywords:** AKT, glucose metabolism, hepatocellular carcinoma, miR‐23a, STAT5A, tumor growth

## Abstract

Hepatocellular carcinoma (HCC) is one of the most common malignancies worldwide. Here, we identified that increased miR‐23a expression in HCC tissues was associated with worse survival. More importantly, we found that STAT5A was a target of miR‐23a, whose levels significantly decreased in tumor tissues. Stable expression of STAT5A in Huh7 cells suppressed glucose metabolism and tumor growth. Finally, this study showed that increased miR‐23a negatively regulated STAT5A, which further activated AKT signaling to enable rapid metabolism for accelerated tumor growth in HCC. Taken together, our results demonstrated that the miR‐23a‐STAT5A‐AKT signaling pathway is critical to alter glucose metabolism in HCC and may offer new opportunities for effective therapy.

## Graphical abstract

Hepatocellular carcinoma (HCC) is one of the leading causes of cancer deaths worldwide. Metabolic reprogramming has been recognized as a core feature in HCC. In line with this idea, we verified that increased miR‐23a inhibited STAT5A expression and promoted glucose metabolism through activated AKT phosphorylation in HCC. This new signaling pathway may offer new treatment target for HCC patients.

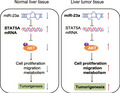

**Abbreviations**
GC/MS: gas chromatography/mass spectrometry
HCC: hepatocellular carcinoma
PVTT: portal vein tumor thrombus
STAT5A: Signal Transducer and Activator of Transcription 5A

## 1 Introduction

Hepatocellular carcinoma (HCC) is one of the most common malignancies worldwide [1] and fourth most commonly diagnosed cancers among Chinese men [2]. Although progress in understanding, diagnosing, and treating patients with HCC has been made in the past decade, the outcome of treatments remains unsatisfactory because of frequent recurrence and drug resistance [3, 4]. In recent years, there has been a growing interest in understanding cancer metabolism, especially the reprogramming of glucose metabolism in cancer cells. Moreover, therapeutic potential of selectively targeting glucose metabolisms in cancer cells has been exploited [5].

MicroRNAs (miRNAs) are reported to play various roles in HCC [6]. miRNA‐34a promotes tumor metastasis in HBV‐positive HCC patients [7]; decreased miR‐199a/b‐3p in HCC suppresses HCC growth by inhibiting PAK4/Raf/MEK/ERK signaling pathway [8]. miR‐23a is also aberrantly expressed in several human cancers as reported before [9, 10, 11, 12]. miR‐23a can regulate proliferation in prostate cancer cells by targeting sinomenine [13]. Besides, enhanced glutaminase and glutamine metabolism can be observed by suppression of miR‐23 in PC3 cells [14]. However, a single microRNA is able to regulate multiple mRNAs according to some database (microRNA.org, TargetScan, microRNASeq). Other potential targets and signaling of miR‐23a in HCC are not clear.

Previous evidence also demonstrates that miR‐23a is upregulated in HCC and suppresses gluconeogenesis through STAT3 and PGC‐1α/G6PC [15]. Signal Transducer and Activator of Transcription 5 (STAT5), another member of the STAT family (STAT1, STAT2, STAT3, STAT4, STAT5A, STAT5B, STAT6), can be activated by many cytokines [16, 17, 18] and detected in many solid tumors [19, 20, 21, 22]. There are two STAT5 isoforms, STAT5A and STAT5B, which are encoded by two interconnected genes on chromosome 17 [23]. Although both STAT5 isoforms are roughly 95% homologous at the level of cDNA, they present different contributions to carcinogenesis. Notably, loss of STAT5 signaling results in hepatic steatosis through elevated STAT1/STAT3 activity [24, 25] and CD36 [26]. STAT5A also acts as a key tumor suppressor by reciprocally inhibiting expression of NPM1‐ALK [27]. However, the precise role of STAT5A in HCC to glucose metabolism is still not clear.

In this study, we found that miR‐23a expression was upregulated in HCC, while its target, STAT5A, was greatly reduced and associated with poor RFS and OS. Besides, we verified that increased STAT5A inhibited cell proliferation *in vitro* and *in vivo* and also reduced glucose consumption and lactate production. Using a metabolic flux analysis based on GC/MS platform, we demonstrated that STAT5A was important in metabolic reprogramming of glucose metabolism *in vitro* and *in vivo*. Importantly, we revealed that miR‐23a‐STAT5A‐AKT signaling was responsible for this changed glucose metabolism in HCC and this axis may offer new chance for effective therapy.

## 2 Materials and methods

### 2.1 Cell culture

The cells were purchased from the Chinese Academy of Science Cell Bank. All cells were cultured in Dulbecco's modified Eagle's medium (DMEM, Hyclone, Logan, UT, USA) supplemented with 10% fetal bovine serum (FBS, Hyclone) and 1% penicillin–streptomycin (Gibco, New York, NY, USA), in an atmosphere of 95% air and 5% CO_2_.

### 2.2 Tissue microarray

All HCC human specimens were obtained after hepatectomy. The patients provided written informed consent before the surgery according to the rules and regulations of our institution. Immunohistochemistry was performed using the STAT5A antibody (Abcam, Cambridge, MA, USA, ab32043). Horseradish peroxidase–conjugated anti‐rabbit secondary antibodies were then applied. Score of the tissue microarray was performed with aperio imagescope v12.0.1.5027 (Leica Biosystems, Buffalo Grove, IL, USA).

### 2.3 RNA interference

2 × 10^5^ cells were seeded in a 6‐well plate first. After 24 h of adherent culture, siRNA/mi RNA was transfected into cells by Lipofectamine 2000 Transfection Reagent (Invitrogen, Carlsbad, CA, USA) and lasted for 48 h. Then, cells were lysed using cell lysis buffer in order to perform western blot measurement. The siRNA sequences are listed as follows. siRNA‐STAT5A: sense 5′‐GCUGGCUAAAGCUGUUGAUdTdT‐3′, anti‐sense 5′‐AUCAACAGCUUUAGCCAGCdTdT‐3′. hsa‐miRNA 23a mimics: sense 5′‐AUCACAUUCCAGGGAUUCC‐3′, anti‐sense 5′‐AAAUCCCUGGCAAUGUGAUUU‐3′. hsa‐miRNA 23a inhibitor: 5′‐GGAAAUCCCUGGCAAUGUGAU‐3′.

### 2.4 Cell proliferation and cell cycle

For the proliferation assays, cells were seeded into 96‐well plates (2000 cells per well) at the same time. Cell viability was determined every 24 h using a Cell Counting Kit‐8 (OBIO Cell Counting Kit, OCPA(C) 2012001, Obio Technology, Shanghai, China), according to the manufacturer’s instructions. For cell cycle analysis, both floating and adherent cells were collected and washed with cold phosphate‐buffered saline (PBS) and fixed with 70% ethanol overnight at 4 °C. Cells were then treated with staining buffer (PBS containing 1 mg·mL^−1^ PI and 10 mg·mL^−1^ RNaseA (Beyotime, C1052, Shanghai, China) at 37 °C in the dark for 30 min. The samples were analyzed with a flow cytometer (Beckman, Quanta SC).

### 2.5 Cell migration assays

Using Trans‐well Permeable Supports (Corning, New York, NY, USA), 1 × 10^4^ cells were suspended in DMEM (FBS‐free) and plated in the top chamber. DMEM containing 10% FBS was added to the bottom chamber. After 48 h, migratory cells in the bottom chamber were stained by crystal violet, and cell number was pictured (Olympus TH4‐200, Tokyo, Japan) and counted by Adobe Photoshop CS4.

### 2.6 Western blot

Cells were lysed in ice‐cold lysis buffer and EDTA‐free protease inhibitors (Biotool, Switzerland). In western blotting experiments, proteins were separated on 10% SDS/polyacrylamide gels, and transferred to poly(vinylidene fluoride) (PVDF, Millipore, Burlington, MA, USA) membranes. Antibodies included STAT5A (Abcam, ab32043), and STAT5A phospho Y694 (Abcam, ab30648), p‐AKT (S473) (CST, 4060S, Danvers, MA, USA), p‐AKT (T308) (CST, 13038S), GAPDH (Proteintech, 60004‐1‐Ig), and AKT (Proteintech, 10176‐2‐AP).

### 2.7 Luciferase activity

Huh7 cells were seeded into 48‐well plates and were cotransfected with a mixture of 200 ng of firefly luciferase reporter, 10 ng of pRL‐CMV Renilla luciferase reporter, and miR‐23a mimics or inhibitor (20 nm). After 48 h, the firefly and Renilla luciferase activities were measured with a dual‐luciferase reporter assay (Promega, Madison, WI, USA).

### 2.8 RNA fluorescence in situ hybridization (FISH)

The detailed steps of FISH were carried out as reported [28]. Oligonucleotide modified probe sequence for human miRNA 23a. The analysis software Image‐Pro plus 6.0 (Media Cybernetics, Inc, Rockville, MD, USA) was applied to acquire the Immunofluorescence Accumulation Optical Density (IOD) for evaluating the expression of miRNA 23a in HCC tissues.

### 2.9 Immunohistochemistry

IHC was performed according to the previous report [29]. According to the intensity of staining (0: no staining, 1: weak staining, 2: moderate staining, and 3: strong staining) and the percentage of stained cells (0: 0%, 1: 1–24%, 2: 25–49%, 3: 50–74%, and 4: 75–100%), each specimen was assigned a final score determined by multiplying the intensity score with the percentage score (scored as 0, 1, 2, 3, 4, 6, 8, 9, and 12). Antibodies included STAT5A (Abcam, ab32043), p‐AKT (S473) (CST, 4060S), and p‐AKT (T308) (CST, 13038S).

### 2.10 Glucose consumption and lactate production

2 × 10^6^ cells were seeded in a plate first. After 24 h of adherent culture, the amounts of glucose and lactate present in the medium were tested by the D‐Glucose Enzymatic Bio‐Analysis Kit from R‐Biopharm (Cat.No.10716251035) and the L‐Lactic acid Enzymatic Bio‐Analysis Kit from R‐Biopharm (Cat.No.10139084035), respectively.

### 2.11 Metabolic flux experiments using [U‐^13^C_6_]‐glucose

The medium used for uniformly labeled [U‐^13^C_6_]‐glucose experiments composed of low‐glucose DMEM (Gibco 11054‐020: 1 g·L^−1^ glucose, no glutamine) with supplement of 1 g·L^−1^ [U‐^13^C_6_] glucose, 10% (v/v) fetal bovine serum, the 1 mm pyruvate, the unlabeled 2 mm L‐glutamine, and 1% (v/v) penicillin–streptomycin (Gibco). The medium was adjusted to pH 7.0–7.4 and filtered sterilization for usage. Cells were seeded at a density of approximately 2 × 106 cells per 10 cm dish. Labeling medium was used to replace the unlabeled medium when cells grew up to ~ 60 % confluence. At this point, it was regarded as *t* = 0 h. Cell samples at 12 or 24 h were collected. In brief, we collected cell samples and extracted metabolites as follows: We quickly aspirated the medium completely and immediately, put plates on dry ice, and added 2 mL of 50% (vol/vol) methanol (cooled to −80 °C) to incubate for 20 min on dry ice. Next, we scraped the plates on the dry ice with cell scraper and transferred the cell lysate/methanol mixture to a 5‐mL tube on dry ice, and centrifuged the tube at 14 000 ***g*** for 5 min at 4–8 ℃, and again transferred the metabolite‐containing supernatant to a new 5‐mL tube on dry ice. After adding 500 μL 50% (vol/vol) methanol to the pellet in 5‐mL tubes and vortexed for 1 min at 4–8 °C, we spun the tubes at 14 000 ***g*** for 5 min at 4–8 °C, and then transferred the supernatant to the above 5‐mL supernatant tube together, and mixed and divided 90% of total extractions to new 1.5‐mL tubes for metabolite measurement. About 10% of total volume was transferred to another new tube for protein concentration determination for normalization. Finally, we used speed Vac to lyophilize to a 1.5 mL pellet using no heat and stored them at −80 °C for analysis.

### 2.12 Labeled metabolites of isotopomer measurement by GC/MS

Nonsugar metabolites were derivatized for GC/MS analysis and followed a previously published protocol [30]. First, 70 μL of 20 mg·mL^−1^ O‐isobutylhydroxylamine hydrochloride (TCI) was added to the dried pellet and incubated for 20 min at 80 °C. After cooling, 30 µL N‐tert‐butyldimethylsilyl‐N‐methyltrifluoroacetamide (Sigma, Darmstadt, Germany) derivatizing agent was added and samples were reincubated for 60 min at 80 °C before centrifugation for 10 min at 18 000 ***g*** (4 °C). The supernatant was transferred to an autosampler vial for GC/MS analysis. A Shimadzu QP‐2010 Ultra GC/MS was programmed with an injection temperature of 250 °C injection split ratio 1/10 (depending upon sample concentration) and injected with 1 µL of sample. GC oven temperature started at 110 °C for 4 min, rising to 230 °C at 3 °C·min^−1^ and to 280 °C at 20 °C·min^−1^ with a final hold at this temperature for 2 min. GC flow rate with helium carrier gas was 50 cm·s^−1^. The GC column used was a 30 m × 0.25 mm × 0.25 mm HP‐5ms. GC/MS interface temperature was 300 °C, and (electron impact) ion source temperature was set at 200 °C, with 70 V/150 µA ionization voltage/current. The mass spectrometer was set to scan *m*/*z* range 50–800, with 1 kV detector. GC/MS data were analyzed to determine isotope labeling and quantities of metabolites. Metabolites with baseline separated peaks were quantified on the basis of total ion count peak area, using standard curves generated from running standards in the same batch of samples. To determine 13C labeling, the mass distribution for known fragments of metabolites was extracted from the appropriate chromatographic peak. These fragments either contained the whole carbon skeleton of the metabolite, or lacked the alpha carboxyl carbon, or (for some amino acids) contained only the backbone minus the side chain [31]. For each fragment, the retrieved data comprised mass intensities for the lightest isotopomer (without any heavy isotopes, M0) and isotopomers with increasing unit mass (up to M6) relative to M0. These mass distributions were normalized by dividing by the sum of M0 to M6 and corrected for the natural abundance of heavy isotopes of the elements H, N, O, Si, and C, using matrix‐based probabilistic methods as described [32], and implemented in MATLAB [33]. Labeling results are expressed as average fraction the particular compound that contains isotopic label from the particular precursor.

### 2.13 Animals

Xenografts derived from Huh7 were subcutaneously implanted in 4‐week‐old male nude mice and were randomly separated into groups. Tumor volume was estimated using the formula 0.52*length*width^2^. All procedures were in agreement with the guidelines for the Care and Use of Laboratory Animals and were approved by the Animal Care and Use Committee, Shanghai Institutes for Biological Sciences.

### 2.14 Statistics analysis

Statistical analyses were performed with spss version 21.0 software (IBM, New York, NY, USA). Other experiments of significant differences between groups were determined using the Student’s *t* test (two‐tailed) by graphpad prism 8.0 software (San Diego, CA, USA). The investigators were totally blinded to group allocation during data collection and analysis.*P < 0.05, **P < 0.01, ***P < 0.001.

All other materials and methods are described in the supplementary information.

## 3 Results

### 3.1 miR‐23a is highly expressed and associated with poor survival in HCC patients

**Fig. 1:**
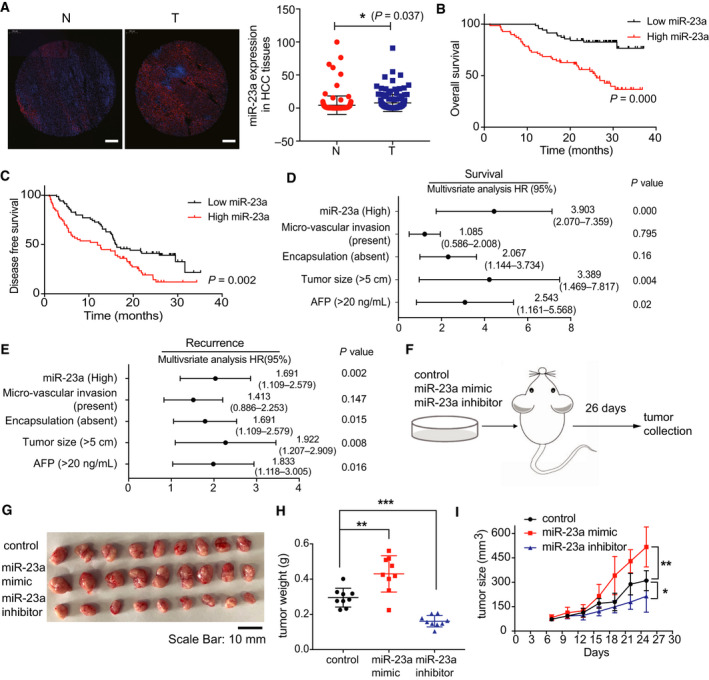
miR‐23a upregulation correlated with poor survival and prognosis in HCC patients. (A) Representative miR‐23a expression measured by FISH and its quantification in matched 140 HCC patients’ normal (N) and tumor tissues (T) (Scale bar, 200 μm). (B, C) Overall and disease‐free survival curve based on miR‐23a expression in 140 patients. (D, E) Multivariate analysis of the hazard ratios (HRs) based on miR‐23a expression in HCC patients’ survival and recurrence. (F) Experimental design of xenograft model in nude mice. (G) Representative images of xenograft tumors after injection of miR‐23a mimic and inhibitor (*n* = 9). (H)Tumor weight analysis of g (*n* = 9). (I)Tumor growth curve in xenograft mouse model after transfection of miR‐23a mimic and inhibitor (*n* = 9). Kaplan–Meier method was used to estimate survival curves, which were compared using the log‐rank test. SPSS was used for statistics analysis in multivariate analysis. All results are presented as mean ± SD. *P < 0.05; **P < 0.01; ***P < 0.001.

To evaluate the potential of miR‐23a as a prognostic indicator, we first checked the expression of miR‐23a in HCC tissues by fluorescence in situ hybridization (FISH). Results demonstrated that miR‐23a importantly increased in human tumor tissues (Fig. 1A). As listed in Table S1, high miRNA 23a expression was associated with high total bilirubin (TB) level and high portal vein tumor thrombus (PVTT) occurrence rate. The Kaplan–Meier survival test was then performed. It was shown that high levels of miR‐23a were associated with poor overall survival (OS) (*P* < 0.001) and disease‐free survival (DFS) (*P* = 0.002) rates (Fig. 1B,C). Multivariate analyses further revealed that increased miR‐23a expression, together with encapsulation, big tumor size, and high AFP level, was independent risk factors for OS and DFS (Fig. 1D,E). To address the functional role of enhanced miR‐23a in HCC growth, miR‐23a mimic and miR‐23a inhibitor were transfected into Huh7 cells first for 48 h (Fig. 1F). Xenograft mouse model verified that increased miR‐23a could promote tumor growth (Fig. 1G,H), while inhibition of miR‐23a inhibited tumor formation. Taken together, these data revealed that miR‐23a was associated with patients' survival and played oncogenic role in HCC progression.

### 3.2 STAT5A is a direct target of miR‐23a

**Fig. 2:**
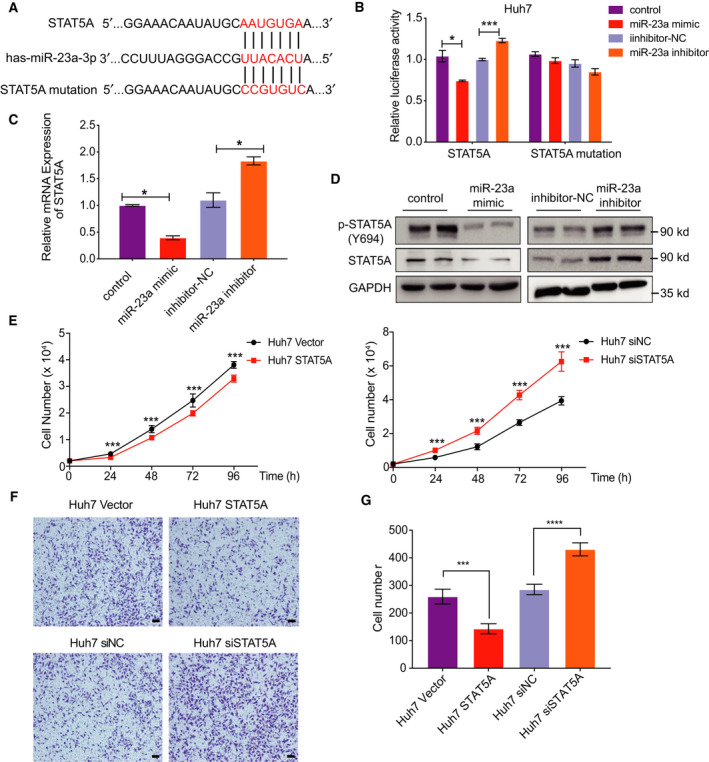
STAT5A, target of miR‐23a, influenced tumor proliferation and migration in HCC. (A) miR‐23a binding sites in STAT5A 3'UTR. (B) Relative luciferase activity of STAT5A or STAT5A mutation plasmids with miR‐23a mimic or inhibitor cotransfection in Huh7 cells (*n* = 6). (C, D) RNA and protein level of STAT5A regulated by miR‐23a (*n* = 3). (E) Cell proliferation with or without STAT5A expression in Huh7 cells (*n* = 6). (F, G) Cell migrations with or without STAT5A expression in Huh7 cells (*n* = 3) (Scale bar, 20 μm). All results are presented as mean ± SD. *P < 0.05; **P < 0.01; ***P < 0.001.

By searching online database library, we found numerous potential target proteins, which may have miR‐23a binding sites within 3' UTR. STAT5A was one of these candidate targets according to bioinformatic analyses (microRNA.org, TargetScan, microRNASeq). To verify this hypothesis, STAT5A 3' UTR, containing miR‐23a binding site or mutated miR‐23a binding site, was cloned downstream of the luciferase open reading frame (Fig. 2A). The luciferase constructs were transfected into Huh7 cells with miR‐23a mimic or miR‐23a inhibitor, respectively. Altered expression of luciferase activity was observed in the STAT5A group, but not in STAT5A mutant 3' UTR groups (Fig. 2B). Using qRT‐PCR and western blotting analyses, we also confirmed that miR‐23a controlled the expression of STAT5A negatively (Fig. 2C,D). We then studied the biological functions of STAT5A in Huh7 cells. Growth curves demonstrated that overexpressed STAT5A could inhibit cell proliferation and STAT5A knockdown could increase cell proliferation *in vitro* (Fig. 2E). Consistently, flow cytometry results suggested that the percentage of cells in G0/G1 phase significantly increased in the high STAT5A group compared with the control group, while percentages in the G2/M phase decreased (Fig. S1A–D). Additionally, increased STAT5A also suppressed cell migration (Fig. 2F,G). Of note, our study is the first to suggest that STAT5A is a direct target of miR‐23a, which is critical for cell proliferation, cell cycle, and migration.

### 3.3 Loss of STAT5A contributes to poor prognosis in HCC patients

**Fig. 3:**
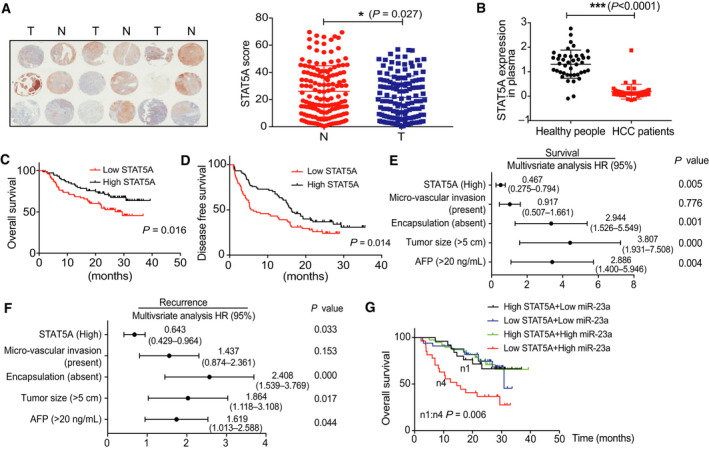
Decreased STAT5A expression correlated with poor survival and prognosis in HCC patients. (A) Representative tissue microarray image and STAT5A expression score in matched 148 HCC patients’ liver tissues. (B) Expression of STAT5A in plasma (*n* = 42). (C, D) Kaplan–Meier overall survival curve and disease‐free survival of patients based on STAT5A expression in 148 patients. (E, F) Multivariate analysis of the hazard ratios (HRs) based on STAT5A expression in HCC patients’ survival and recurrence, together with encapsulation, tumor size, and AFP level. (G) Kaplan–Meier survival curve based on the combination of STAT5A and miR‐23a (*n* = 123). Kaplan–Meier method was used to estimate survival curves, which were compared using the log‐rank test. SPSS was used for statistics analysis in multivariate analysis. All results are presented as mean ± SD. *P < 0.05; **P < 0.01; ***P < 0.001.

Considering the role of STAT5A in HCC is controversial, we first checked the protein level of STAT5A in paired nontumor adjacent tissues (N) and tumors tissues (T) by IHC staining including 148 HCC specimens (Fig. 3A). Other clinicopathological characteristics are shown in Table S2. According to the scores evaluated by the staining‐positive cells, we found that STAT5A expression was sharply reduced in tumor tissues compared with nontumor adjacent tissues. This decline was also found in patients' plasma (Fig. 3B). Besides, as shown in the Kaplan–Meier survival curves, patients with high expression of STAT5A received a much better OS (*P* = 0.016) and DFS (*P* = 0.014) (Fig. 3C,D). Multivariate Cox regression analysis demonstrated that decreased STAT5A expression, together with encapsulation, big tumor size, and high AFP level, was an independent factor associated with RFS and OS (Fig. 3E,F). To determine whether the combination of miR‐23a and STAT5A was a more accurate prognostic factor, patients were classified into four groups according to their miR‐23a and STAT5A expression. Patients in group 4 (low STAT5A + high miR‐23a) received a worst OS (Fig. 3G). Therefore, our results suggest that STAT5A was significantly downregulated in liver tumor tissues and may serve as a potential prognosis biomarker in HCC.

### 3.4 Overexpressed STAT5A represses glucose metabolism and tumor growth in HCC

**Fig. 4:**
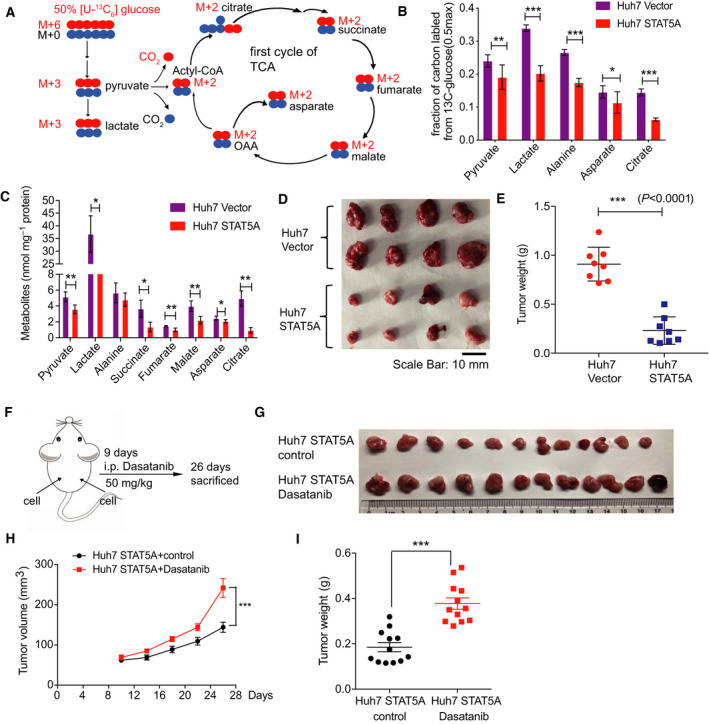
STAT5A inhibited glucose metabolism and tumor growth. (A) Schematic diagram of the fate of [U‐^13^C_6_]‐glucose in glycolysis and TCA cycle. (B, C) Fraction of carbon labeled from the [U‐^13^C_6_]‐glucose and metabolite quantification in Huh7‐Vector and Huh7‐STAT5A cells (*n* = 3). (D, E) Tumor images and tumor weight in xenograft mouse model injected with Huh7‐Vector and Huh7‐STAT5A cells (2 × 10^6^ cells) in nude mice (*n* = 8). (F) Experimental design of xenograft model with or without dasatinib injection. (G–I) Inhibiting STAT5A by Dasatinib promotes tumor growth *in vivo*. All results are presented as mean ± SD. *P < 0.05; **P < 0.01; ***P < 0.001.

Increasing evidences between signaling pathways and metabolic activities are reported, and the importance of metabolic reprogramming in cancers is also widely recognized. To test whether STAT5A influences glucose metabolism, we first measured glucose uptake and lactate secretion in STAT5A overexpression cells. Results confirmed that overexpressed STAT5A decreased glucose uptake and lactate product importantly (Fig. S2A,B). What's more, to explore the detailed changes during glucose metabolism, we used stable isotope‐labeled [U‐^13^C_6_] glucose to track metabolic flux and quantify metabolite production in cells (Fig. 4A).

We observed that the ratio of enriched labeled carbon of metabolites including lactate, pyruvate, and alanine from labeled [U‐^13^C_6_]‐glucose decreased apparently in over‐STAT5A group, suggesting that overexpressed STAT5A suppressed glycolysis rate (Fig. 4B). Similarly, the fraction of labeled carbon of citrate and aspartate in TCA pathways (Fig. 4B) also clearly reduced, implicating a slowed TCA metabolism. Farther quantification of cellular intermediate metabolites in glycolysis and TCA also demonstrated that overexpressed STAT5A reduced lactate, pyruvate, succinate, and citrate about 3 times (Fig. 4C), which was agreed with the labeled results that STAT5A overexpression inhibited glycolysis and TCA metabolism in HCC. Indeed, this weakened glucose metabolism inhibited tumor growth *in vivo* as we expected (Fig. 4D,E). To further confirm this founding, we use dasatinib (Selleck, S1021) as a STAT5A inhibitor (Fig. S2C). In line with these results, we implanted Huh7 cells in nude mice and then randomly separated mice into two groups. Dasatinib (Selleck, S7782) was injected intraperitoneally (50 mg·kg^−1^) [34] when we measured tumor volume every 3 days compared with PBS in the control group. A faster tumor growth was observed in nude mice treated with dasatinib (Fig. 4F–I). Thus, these data demonstrate that STAT5A can attenuate glucose metabolism and tumor growth in HCC.

### 3.5 Decreased STAT5A induces AKT phosphorylation and accelerates cell metabolism in HCC

**Fig. 5:**
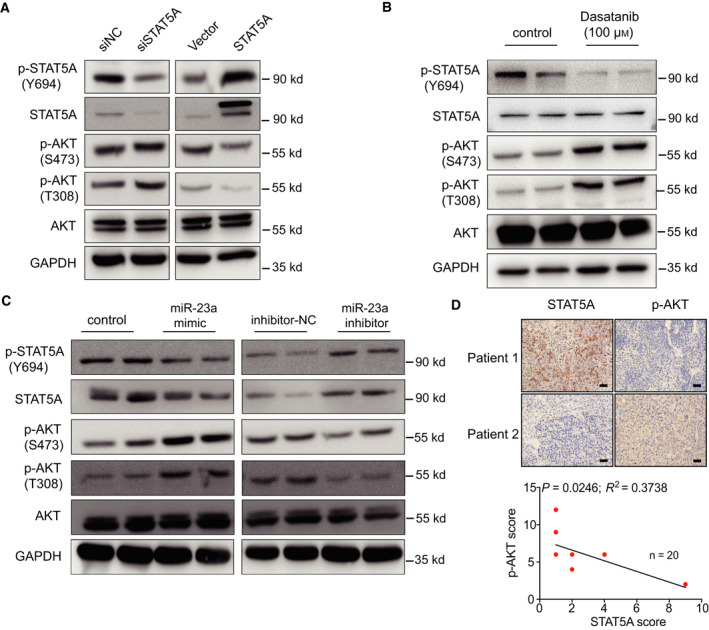
miR23a inhibited STAT5A expression negatively via enhancing AKT phosphorylation. (A, B) Genetic perturbance of STAT5A expression affected AKT phosphorylation in Huh7 cells. (C) miR‐23a mimic and inhibitor transfection affected AKT phosphorylation in Huh7 cells. (D) Representative images and negative correlation between STAT5A and p‐AKT expression in 20 HCC patients (Scale bar, 25 μm). All results are presented as mean ± SD. *P < 0.05; **P < 0.01; ***P < 0.001.

Next, we set out to elaborate the mechanisms of how loss of STAT5A expression promoted glucose metabolism and tumor growth in HCC. Study demonstrates that Akt is a ‘Warburg kinase’, which is important for enhanced cellular energy metabolism and oncogenesis [35]. Surprisingly, enhanced AKT phosphorylation was observed when STAT5A was knocked down genetically (Fig. 5A), which could explain the phenotypes presented above. Notably, inhibiting only p‐STAT5A expression by dasatinib, p‐AKT level still increased significantly (Fig. 5B). Besides, after transfecting miR‐23a into Huh7 cells, a positive regulation was also found between miR‐23a and p‐AKT (Fig. 5C). To further corroborate this relationship, we checked the expression of STAT5A and p‐AKT in 20 HCC specimens by IHC and also revealed a same negative correlation between STAT5A and p‐AKT (*P* = 0.0246) (Fig. 5D). Thus, our findings provide evidence that loss of STAT5A leads to AKT activation and promotes cellular metabolism and tumor growth in HCC.

### 3.6 Inhibiting AKT phosphorylation attenuates increased glucose metabolism and tumor growth induced by loss of STAT5A

**Fig. 6:**
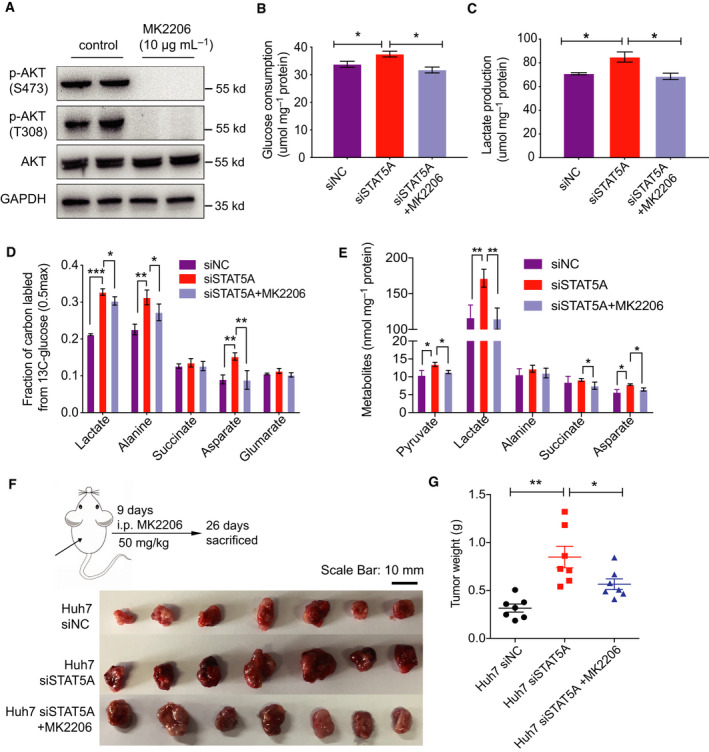
Inhibiting AKT phosphorylation by MK2206 attenuated STAT5A knockdown‐induced metabolism increasement and tumor growth. (A) AKT inhibitor MK2206 inhibited AKT phosphorylation. (B, C) Glucose consumption and lactate production after STAT5A knockdown or combination with MK2206 (*n* = 3). (D, E) Metabolite quantification and fraction of carbon labeled from the [U‐^13^C_6_]‐glucose after STAT5A knockdown or combination with MK2206 (*n* = 3). (F, G) Tumor images and tumor weight in xenograft mouse model injected with siSTAT5A and combination with MK2206 in nude mice (*n* = 8). All results are presented as mean ± SD. *P < 0.05; **P < 0.01; ***P < 0.001.

**Fig. 7:**
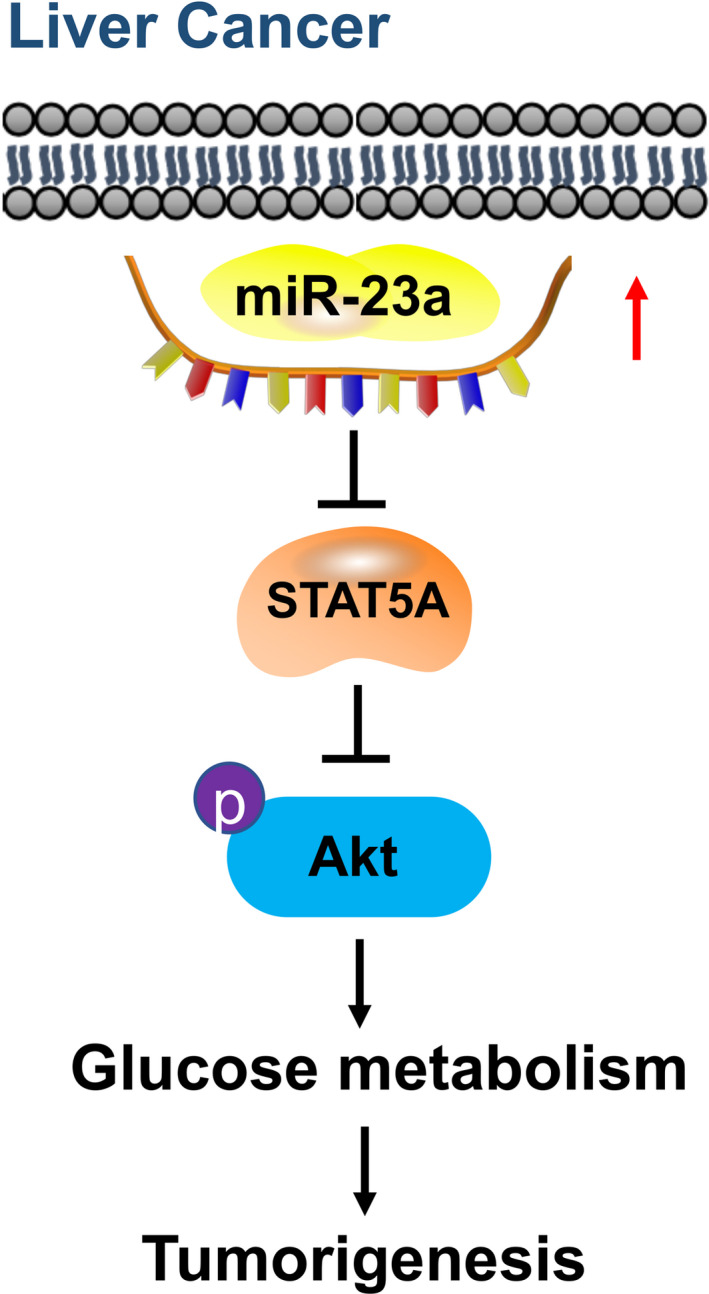
Schematic representation of the proposed mechanism. In liver tumor tissue, miR‐23a upregulation inhibited STAT5A expression, leading to increased AKT phosphorylation to promote cell metabolism and tumorigenesis.

To evaluate loss of STAT5A accelerates glucose metabolism and HCC via AKT activation, we inhibited AKT phosphorylation by MK2206 (Fig. 6A) when STAT5A was knocked down and measured metabolic flux and tumor growth *in vivo*. Results demonstrated that knockdown of STAT5A expression promoted glycolysis and TCA metabolism (Fig. 6B–E), while inhibiting AKT successfully reversed this higher glucose metabolism (Fig. 6B–E). Besides, after constructing siSTAT5A cells in dishes with siRNA for 48 h, similar results were also observed that tumor growth was inhibited significantly by MK2206 compared with the STAT5A knockdown group (Fig. 6F,G). Taken together, as shown in Fig. 7, in normal liver tissue, low miR‐23a expression accelerates STAT5A expression, and AKT cannot be excessively activated. However, in liver tumor tissue, high expression of miR‐23a contributes to a decreased expression of STAT5A. Furthermore, STAT5A subsequently induced AKT phosphorylation, which resulted in enhanced tumor glucose metabolism and tumor growth in HCC. Our research suggests that miR‐23a and STAT5A may serve as potential diagnosis markers and targeting miR‐23a‐STAT5A‐AKT signaling may offer new treatment choices for HCC patients.

## 4 Discussion

In this present work, we found that miR‐23a was aberrantly expressed in HCC and could serve as a promising prognostic factor for patients. Meanwhile, to our knowledge, it was also the first study that revealed that STAT5A was a direct target of miR‐23a. Furthermore, we confirmed that increased miR‐23a promoted glucose metabolism and tumor growth through miR‐23a‐STAT5A‐AKT signaling in HCC.

MicroRNAs (miRNAs) play important roles in HCC progression [36]. Our previous study suggested that the activation of the TGF‐β‐miR‐34a‐CCL22 axis could promote HCC cells disseminated into the portal venous system through the creation of an immune‐subversive microenvironment [7]. Besides, another experiment revealed that increased miR‐135a facilitated PVTT formation by targeting metastasis suppressor 1 (MTSS1) [37]. Here, in this study, we also found that miR‐23a sharply increased and high expression of miR‐23a could contribute to poor survival in HCC patients. Increased miR‐23a accelerated tumor growth *in vitro* and *in vivo*. miR‐23a has been widely reported in different cancers. c‐Myc enhances mitochondrial glutaminase and glutamine metabolism by suppressing the expression of miR‐23 [14]. miR‐23a is also essential for CD4+ T‐cell function [10]. On the other hand, miR‐23a in HCC leads to decreased glucose production, which directly targets PGC‐1α and G6PC [15]. Another recent article shows that miR‐23a from exosomes regulates PD‐L1 expression in macrophages to help HCC cells escaping from immune system [38]. As a particular miRNA has various potential targets, we first reported that STAT5A was a direct target of miR‐23a.

Evidences have revealed that STAT5 is important in cell growth, cell cycle, lipid metabolism, hematopoiesis, host defense, and immunoregulation in HCC [20, 27, 39, 40]. Due to differences in the transactivation domain, STAT5A and STAT5B, two isoforms of STAT5, exhibit various and different oncogenic functions. STAT5B deficiency can result in immunodeficiency and growth failure [41, 42]. Specifically, STAT5B is important for the FOXP3 and interleukin‐2Rα (CD25) [43, 44], which are responsible for the differentiation of regulatory T cells. At the same time, STAT5A also contributes to cell cycle. Cdkn2b and NPM1‐ALK are under the control of STAT5A [27, 45]. Therefore, in this study, we first confirmed the expression of STAT5A in HCC tissues and explored the relationship between STAT5A expression and patients' survival. Results showed that decreased expression of STAT5A resulted in poor patients' survival. On the other hand, function roles explored *in vitro* and *in vivo* suggested that STAT5A could inhibit tumor growth and migration. The importance of metabolic reprogramming in cancers is being increasingly recognized. In many cancers, increased glucose uptake exists to support the rapid cell growth and proliferation [46]. Moreover, cancer cells also have increased glutamine uptake and glutaminolysis, which, in turn, replenish mediate production of the tricarboxylic acid (TCA) cycle [47]. Compatible with previous reports that miR‐23a influenced glucose metabolism in cancers, we observed that over‐STAT5A could importantly restrict glucose metabolism especially the glycolysis using stable isotope‐labeled [U‐^13^C_6_] glucose tracking.

Mechanistically, in this study, we found that STAT5A also negatively mediated AKT phosphorylation. This result was confirmed in common liver cell lines and human tissues. Increasing evidence showed that activation of AKT contributes to enhanced glucose uptake [48], which can explain the phenotype we found. To verify the STAT5A‐AKT pathway, we added AKT inhibitor into cells on the basis of siSTAT5A. Results demonstrated that we were able to reverse the phenotype induced by knockdown of STAT5A. It is reported that activated STAT5 proteins induce activation of the PI3‐kinase/Akt via the Gab2 scaffolding adapter in Ba/F3 cells [49]. However, we did not find the same mechanism in HCC cell lines. What's more, loss of STAT5 signaling led to hepatic steatosis through elevated STAT1/STAT3 activity [24, 25], which was also observed in STAT5A over‐/downexpression cells. Another study also revealed that IL‐6 induced activation of the JAK/STAT3 pathway and also activated the PI3K/AKT and the MEK/ERK pathway [50]. Therefore, we thought that STAT5A may mediate AKT through STAT3 pathway. The exact causality between STAT5A and AKT is not fully understood, and we have established hepatic deletion of STAT5A model to further explore the role of STAT5A in HCC progression.

## 5 Conclusions

In summary, our study identified that miR‐23a was upregulated in HCC, which further inhibited STAT5A expression and promoted tumor growth through activated AKT phosphorylation. miR‐23a‐STAT5A‐AKT pathway may offer new chance for specially targeted and more effective therapy.

## Conflict of interest

The authors declare no conflict of interest.

## Author contributions

YBJ, YZT, HYY, and SQC designed and planned the study. YBJ, YZT, XPZ, and XBW developed and optimized experimental protocols. YBJ, YZT, ML, and XXH performed experiments. BZ and WXG organized patient enrollment, sample collection, and clinical data curation. YBJ, YZT, XPZ, and XBW analyzed and interpreted data. YBJ, YZT, XPZ, XBW, ML, and XXH wrote the manuscript and incorporated feedback from all authors. HYY and SQC provided critical revisions.

## Supporting information


**Fig. S1.** (A–D) Cell cycle analysis with or without STAT5A expression in Huh7 cells. All results are presented as mean ± SD. *P < 0.05; **P < 0.01; ***P < 0.001.

**Fig. S2.** (A–B) Glucose consumption and lactate production in Huh7‐Vector and Huh7‐STAT5A cells (n = 3). (C)Western blot verification of STAT5A inhibition by Dasatinib. All results are presented as mean ± SD. *P < 0.05; **P < 0.01; ***P < 0.001.

**Table S1.** Correlations between clinicopathological characteristics and miRNA 23a expression.

**Table S2.** Correlations between clinicopathological characteristics and STAT5A expression.

## Data Availability

No datasets were generated or analyzed during the current study.
