# Supplementary figures and images for: Loss of STAT5A promotes glucose metabolism and tumor growth through miRNA‐23a‐AKT signaling in hepatocellular carcinoma
Source: Mol Oncol. 2020 Nov 22;15(2):710–24. doi: 10.1002/1878-0261.12846 (PMC7858139; doi:10.1002/1878-0261.12846)

**A**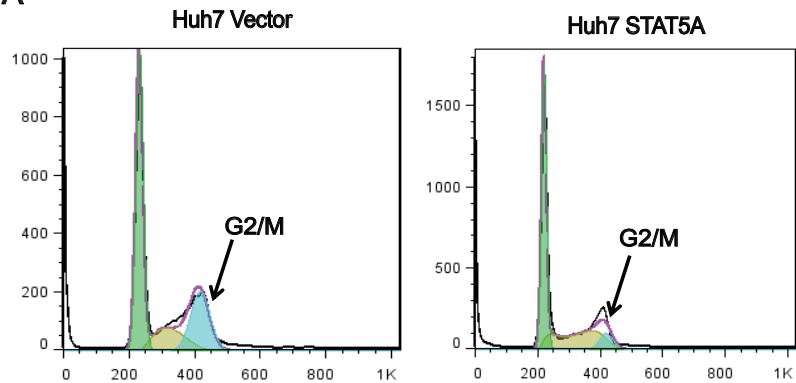**B**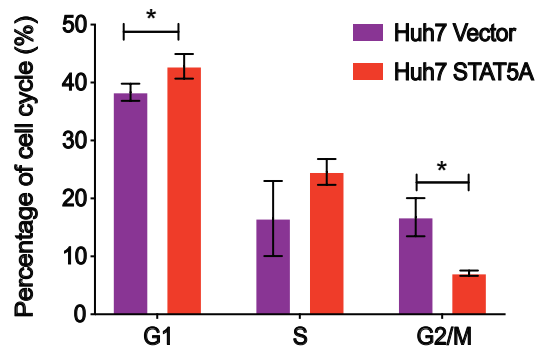**C**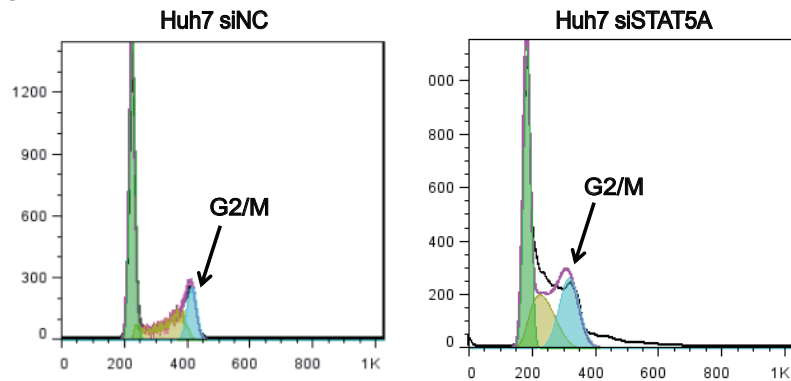**D**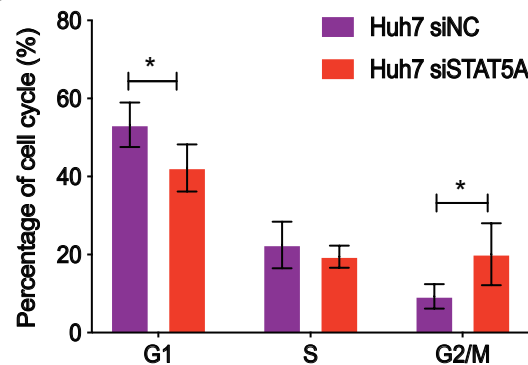

Supplement: Supplementary file 1 — Fig. S1. (A–D) Cell cycle analysis with or without STAT5A expression in Huh7 cells. All results are presented as mean ± SD. *P < 0.05; **P < 0.01; ***P < 0.001. [file MOL2-15-710-s001.pdf]

**A**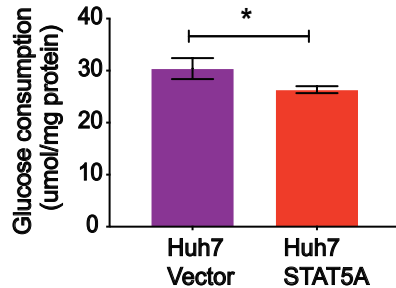**B**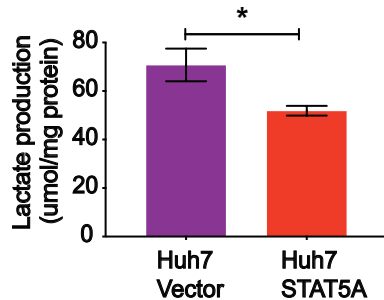**C**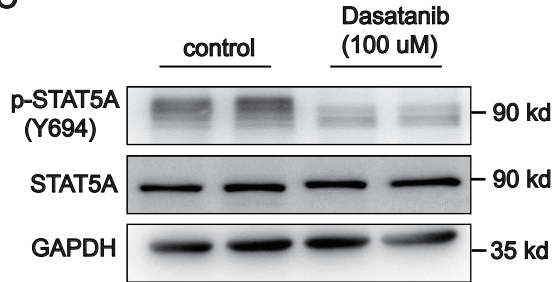

Supplement: Supplementary file 2 — Fig. S2. (A–B) Glucose consumption and lactate production in Huh7‐Vector and Huh7‐STAT5A cells (n = 3). (C)Western blot verification of STAT5A inhibition by Dasatinib. All results are presented as mean ± SD. *P < 0.05; **P < 0.01; ***P < 0.001. [file MOL2-15-710-s002.pdf]
