# Supplementary material for: Loss of STAT5A promotes glucose metabolism and tumor growth through miRNA‐23a‐AKT signaling in hepatocellular carcinoma
Source: Mol Oncol. 2020 Nov 22;15(2):710–24. doi: 10.1002/1878-0261.12846 (PMC7858139; doi:10.1002/1878-0261.12846)
Supplement: Supplementary file 3 — Table S1. Correlations between clinicopathological characteristics and miRNA 23a expression. [file MOL2-15-710-s003.docx]

| **Variables** | **Low(n=70)** | **High(n=70)** | ***P* value** |
| --- | --- | --- | --- |
| **Sex(male/female)**  **Age(years)**  **TB(umol/L)**  **ALB(g/L)**  **ALT(U/L)**  **AST(U/L)**  **AFP(ug/L)**  **HBsAg(positive/negative)**  **HBV DNA load (IU/ML)**  **(≥2000/<2000)**  **Tumor size(cm)**  **Tumor capsule**  **(complete/incomplete)**  **Microvascular invasion**  **(positive/negative)**  **Portal vein tumor thrombus**  **(positive/negative)** | 64/6  47.8±9.4  12.9±4.2  43.2±3.3  43.9±24.0  41.8±25.0  481.6±528.6  62/8  5/65  6.7±3.7  37/33  27/43  4/66 | 62/8  49.7±10.0  14.7±6.0  42.7±5.6  45.7±37.6  46.2±29.2  562.6±570.2  66/4  5/65  6.9±3.5  29/41  30/40  13/57 | 0.573  0.243  **0.033***  0.524  0.740  0.340  0.385  0.365  1  0.624  0.176  0.606  **0.038*** |
