# Supplementary material for: Loss of STAT5A promotes glucose metabolism and tumor growth through miRNA‐23a‐AKT signaling in hepatocellular carcinoma
Source: Mol Oncol. 2020 Nov 22;15(2):710–24. doi: 10.1002/1878-0261.12846 (PMC7858139; doi:10.1002/1878-0261.12846)
Supplement: Supplementary file 4 — Table S2. Correlations between clinicopathological characteristics and STAT5A expression. [file MOL2-15-710-s004.docx]

| **Variables** | **Low(n=74)** | **High(n=74)** | ***P* value** |
| --- | --- | --- | --- |
| **Sex(male/female)**  **Age(years)**  **TB(umol/L)**  **ALB(g/L)**  **ALT(U/L)**  **AST(U/L)**  **AFP(ug/L)**  **HBsAg(positive/negative)**  **HBV DNA load (IU/ML)**  **(≥2000/<2000)**  **Tumor size(cm)**  **Tumor capsule**  **(complete/incomplete)**  **Microvascular invasion**  **(positive/negative)**  **Portal vein tumor thrombus**  **(positive/negative)** | 69/5  47.6±11.0  28.2±83.3  42.7±6.1  50.5±41.5  46.3±29.4  564.3±549.6  70/4  4/70  6.4±3.2  50/24  32/42  16/58 | 67/7  49.6±9.3  14.8±13.2  42.6±3.8  47.9±37.1  45.9±30.0  412.9±517.2  67/7  5/69  6.8±3.9  42/32  25/49  8/66 | 0.547  0.215  0.174  0.908  0.689  0.925  0.087  0.531  1  0.539  0.175  0.237  0.074 |
